# Improving simultaneous saccharification and co-fermentation of pretreated wheat straw using both enzyme and substrate feeding

**DOI:** 10.1186/1754-6834-3-17

**Published:** 2010-08-02

**Authors:** Kim Olofsson, Benny Palmqvist, Gunnar Lidén

**Affiliations:** 1 Department of Chemical Engineering, Lund University, Box 124, SE-221 00 Lund, Sweden

## Abstract

**Background:**

Simultaneous saccharification and co-fermentation (SSCF) has been recognized as a feasible option for ethanol production from xylose-rich lignocellulosic materials. To reach high ethanol concentration in the broth, a high content of water-insoluble solids (WIS) is needed, which creates mixing problems and, furthermore, may decrease xylose uptake. Feeding of substrate has already been proven to give a higher xylose conversion than a batch SSCF. In the current work, enzyme feeding, in addition to substrate feeding, was investigated as a means of enabling a higher WIS content with a high xylose conversion in SSCF of a xylose-rich material. A recombinant xylose-fermenting strain of *Saccharomyces cerevisiae *(TMB3400) was used for this purpose in fed-batch SSCF experiments of steam-pretreated wheat straw.

**Results:**

By using both enzyme and substrate feeding, the xylose conversion in SSCF could be increased from 40% to 50% in comparison to substrate feeding only. In addition, by this design of the feeding strategy, it was possible to process a WIS content corresponding to 11% in SSCF and obtain an ethanol yield on fermentable sugars of 0.35 g g^-1^.

**Conclusion:**

A combination of enzyme and substrate feeding was shown to enhance xylose uptake by yeast and increase overall ethanol yield in SSCF. This is conceptually important for the design of novel SSCF processes aiming at high-ethanol titers. Substrate feeding prevents viscosity from becoming too high and thereby allows a higher total amount of WIS to be added in the process. The enzyme feeding, furthermore, enables keeping the glucose concentration low, which kinetically favors xylose uptake and results in a higher xylose conversion.

## Background

Biomass residues from both forest industry and agriculture, or dedicated perennial (energy) crops, are potential feedstocks for fermentative ethanol production. It is important to use all sugars available, i.e., both hexoses and pentoses, to obtain a high yield. Agricultural materials and hardwoods contain high amounts of pentoses, primarily xylose. Genetic engineering to confer xylose-fermenting abilities to the yeast used in the ethanol industry, *Saccharomyces cerevisiae*, requires the introduction of a pathway converting xylose into xylulose. This can be done by either a one-step isomerization reaction or a two-step reduction-oxidation conversion as described in a number of recent reviews [1-3].

Simultaneous saccharification and fermentation (SSF) [4] has been established as a promising option for ethanol production from lignocellulosic materials [5]. In this process, the enzymatic hydrolysis of the pretreated material takes place together with the fermentation. The overall ethanol yield in SSF has been reported to be higher than if the enzymatic hydrolysis and fermentation are carried out separately (SHF) [6]. However, not only the yield but also the ethanol concentration is important, because the distillation costs decrease as a function of the final ethanol concentration [7]. To increase the ethanol concentration, a high content of water-insoluble solids (WIS) is needed. However, a high WIS content leads to a high viscosity of the medium, leading to severe mixing problems. In practice, there is a maximum WIS content for each feedstock, which can be practically handled in an SSF process. Furthermore, the liquid obtained after pretreatment contains a number of compounds with inhibitory effect on the yeast, and potentially also the enzymes [8]. A high WIS content gives a high concentration of these inhibitors.

Instead of a batch SSF process, one may instead use a fed-batch SSF process. In this way the following advantages are gained: 1) The viscosity of the medium can be maintained low due to a gradual feeding of new material to the reactor, in which the viscosity decreases due to enzymatic degradation [9]; 2) the effect of toxicity of the hydrolyzate can be decreased as a result of both adaptation of the yeast and gradual biological detoxification; 3) there may be a beneficial effect on the xylose uptake from a changed concentration ratio of xylose to glucose in the medium [9-11].

The reason for the last advantage, which is the main focus here, is that even with the heterologous xylose pathway introduced, co-fermentation of xylose and glucose in *S. cerevisiae *is hampered as a result of the inhibition of xylose transport by glucose. The probable main reason for this is that xylose and glucose compete for the same transport systems [12,13], and the affinity for xylose is approximately two orders of magnitude lower than for glucose [14]. Therefore, the glucose concentration must be low to obtain efficient xylose uptake. However, glucose in fact enhances xylose utilization at low but nonzero concentrations [15,16]. This can be attributed to induction of suitable xylose transporter systems [16,17], improved cofactor generation [16] and induction of glycolytic enzymes [18]. A low but nonzero glucose concentration--giving a high xylose to glucose ratio--is therefore desirable in a process aiming at co-fermentation of glucose and xylose.

Recently, several studies on different raw materials have been carried out using simultaneous saccharification and co-fermentation (SSCF) with a genetically engineered strain of *Saccharomyces cerevisiae*, TMB3400, capable of fermenting glucose and xylose [10,19-22]. In these studies, substantial xylose conversions have been reported for various materials such as spruce, corn stover, wheat straw and sugar cane bagasse. After acid-catalyzed steam pretreatment of lignocellulosic materials, the hemicellulosic sugars are mainly present as monomers, whereas a large fraction of the glucose content is present as glucan in the fibers. One main advantage of SSCF in comparison to, e.g., separate hydrolysis and co-fermentation (SHCF) is that the glucose released into the medium by enzymatic hydrolysis is simultaneously fermented, resulting in a low but nonzero glucose concentration in the medium. This is beneficial for the enzymatic hydrolysis in terms of minimizing end-product inhibition, but it also gives a high xylose-to-glucose concentration ratio, which favors xylose uptake.

We have previously shown that by designing the SSCF process such that the glucose concentration in the reactor is kept low, an improved xylose conversion can be obtained. This concept was proven using substrate feeding (fed-batch) for wheat straw [10], prefermentation, i.e. conversion of free glucose before initiating enzymatic hydrolysis, for spruce (*Picea abies*) [21] and controlled enzyme feeding giving a controlled release of glucose in the medium (also for spruce) [22].

In the current work, SSCF of pretreated wheat straw was studied using a combined strategy of substrate and enzyme feeding at relatively high WIS content (starting at 8%, then gradually increasing to 11% due to substrate feed). By substrate feeding, a higher total addition of WIS can be used than in a batch process, and at the same time the enzyme feeding allows a control of glucose concentration. Different enzyme feeding schemes were experimentally investigated, and the fermentation was analyzed in terms of medium composition and product and by-product formation.

## Methods

### Simultaneous saccharification and co-fermentation (SSCF)

#### Raw material and pretreatment

Wheat straw, locally harvested in August 2009 and dried in the field (Johan Håkansson Lantbruksprodukter, Lunnarp, Sweden), was milled and sieved into 1- to 10-mm pieces and soaked overnight in 0.2% (vol/vol) H_2_SO_4 _in room temperature in closed barrels at a solids loading of 10% (wt/wt). The impregnated straw was pressed to 300 bars and reached a dry matter content of 50% and was subsequently steam-pretreated batchwise at 190°C for 10 min in a 10-L reactor. Further description of the equipment is given by Palmqvist et al. [23]. The pretreated material was stored at 4°C. The composition of the pretreatment slurry is shown in Table 1. The water-insoluble and liquid fractions were analyzed using National Renewable Energy Laboratories (NREL) standard procedures [24,25]. The WIS content of the pretreated slurry was measured by washing the fibers with deionized water over filter paper and was determined to be 13% (wt/wt).

**Table 1:** Composition of the pretreated wheat straw material

Content	Material 1	Material 2
Content in solid fraction (% WIS)		
Glucan	54.4	54.4
Xylan	3.1	3.3
Lignin	32.8	31.3
Content in liquid fraction (g L^-1^)		
Glucose^a^	6.7	5.8
Xylose^a^	38.8	36.8
Furfural	4.9	3.1
HMF	0.5	0.2
Acetic acid	3.4	2.5

^a^Both monomeric and oligomeric forms are included.

#### Cell cultivation

The recombinant xylose-fermenting strain *S. cerevisiae *TMB3400 [26] were used in all the fed-batch SSCF experiments. Yeast cells to be used in the SSCF were produced by an initial preculture in shake flask, followed by an aerobic batch cultivation on glucose and finally an aerobic fed-batch cultivation on wheat straw hydrolyzate liquid to improve inhibitor tolerance by adaptation as previously shown by Alkasrawi et al. [27].

The yeast was inoculated in 300-ml flasks containing 100 ml media supplemented with 16.5 g L^-1 ^glucose, 7.5 g L^-1 ^(NH_4_)_2_SO_4_, 3.5 g L^-1 ^KH_2_PO_4_, 0.74 g L^-1 ^MgSO_2_·7H_2_O, trace metals and vitamins. The cells were grown for 24 h at 30°C and pH 5 in a rotary shaker at 180 rpm. Subsequently, aerobic batch cultivations were performed in a 2.5-L bioreactor (Biostat; A. B. Braun Biotech International, Melsungen, Germany) at 30°C. The working volume was 0.7 L, and the medium contained 20.0 g L^-1 ^glucose, 20.0 g L^-1 ^(NH_4_)_2_SO_4_, 10.0 g L^-1 ^KH_2_PO_4_, 2.0 g L^-1 ^MgSO_4_, 27.0 mL L^-1 ^trace metal solution and 2.7 mL L^-1 ^vitamin solution. The cultivation was initiated by adding 20.0 mL of the preculture to the bioreactor. The pH was maintained at 5.0 throughout the cultivation by automatic addition of 3 M NaOH. The trace metal and vitamin solutions were prepared according to the method described by Taherzadeh et al. [28]. Aeration was maintained at 1.2 L min^-1^, and the stirrer speed was kept at 800 rpm. When the ethanol produced in the batch phase was depleted, the feeding of pretreatment liquid from wheat straw was initiated. A quantity of 1.0 L of wheat straw pretreatment liquid was added with an initial feed rate of 0.04 L h^-1^, which was increased linearly to 0.10 L h^-1 ^during 16 h of cultivation. The aeration during the fed-batch phase was maintained at 1.5 L min^-1^, and the stirrer speed was kept at 1000 rpm.

After cultivation, the cells were harvested by centrifugation in 700-mL flasks using a HERMLE Z 513 K centrifuge (HERMLE Labortechnik, Wehingen, Germany). The pellets were resuspended in 9 g L^-1 ^NaCl solution to obtain a cell suspension with a cell mass concentration of 80 g dry wt L^-1^. The time between cell harvest and initiation of the SSCF was no longer than 3 h.

#### SSCF

All fed-batch SSCF experiments were carried out in duplicates under anaerobic conditions using 2.5-L bioreactors (Biostat; A. B. Braun Biotech International, Melsungen, Germany; Biostat A plus; Sartorius, Melsungen, Germany) sterilized by autoclavation. The fed-batch experiments were carried out with a WIS content starting at 8% and gradually increasing to an added total amount corresponding to 11% at a final working broth weight of 1.6 kg. The calculations of the WIS content were based on beginning measurements. To obtain the initially desired WIS content in the bioreactor, the pretreated, undetoxified slurry was diluted with sterile deionized water. Before adding the pretreated slurry to the reactor, pH was adjusted to 4.8 with the addition of 10 M NaOH. All SSCF experiments were carried out at 34°C for 96 h. The pH was maintained at 5.0 throughout the fermentation by automatic addition of 3 M NaOH. The SSCF medium was supplemented with 0.5 g L^-1 ^NH_4_H_2_PO_4_, 0.025 g L^-1 ^MgSO_4_·7H_2_O and 1.0 g L^-1 ^yeast extract. An initial yeast concentration of 4 g dry wt L^-1 ^was used. The enzyme preparations used were Xylanase XL (obtained directly from SAF-ISIS, Souston, France) containing both xylanase and cellulase activity and Novozyme 188 (Novozymes A/S, Bagsvaerd, Denmark). The cellulase activity of Xylanase XL was 44 FPU (filter paper units) g^-1 ^and the β-glucosidase activity of 37 IU g^-1^. Novozyme 188 had a β-glucosidase activity of 342 IU g^-1^. The total amount of enzyme added to respective SSCF experiments corresponded to a cellulase activity of 36 FPU (g total glucan)^-1 ^(i.e., normalized to the total amount of glucan added at the end of the experiment) and a total β-glucosidase activity of 78 IU (g total glucan)^-1^. Samples for high-pressure liquid chromatography (HPLC) analysis were taken repeatedly throughout the SSCF.

### Analysis

The cell mass concentration in the cell suspension (in 9 g L^-1 ^NaCl, described above) was measured in duplicates from 10-mL samples. The samples were centrifuged (1000 × *g*) for 5 min at 3000 rpm (Z200 A; HERMLE Labortechnik, Wehingen, Germany). The supernatants were discarded, and the pellets were washed with 9 g L^-1 ^NaCl solution and centrifuged a second time. The pellets were dried at 105°C overnight and weighed.

FPU activity was determined according to the procedure of NREL [29]. The β-glucosidase activity (1 IU corresponding to conversion of 1 μM substrate min^-1^) measurement method was slightly modified from that of Berghem and Pettersson [30], where *p*-nitrophenyl-β-D-glucoside was used as a substrate. The β-glucosidase cleaves this substrate, forming *p*-nitrophenol, which has an absorbance maximum at 400 nm. The substrate was dissolved in an acetate buffer (0.1 M, pH 4.8), giving a concentration of 5 mM. A quantity of 2 mL of the preheated (50°C) substrate solution was added to a cuvette, and the reaction was initiated by addition of 0.1 mL properly diluted enzyme solution. The production rate of *p*-nitrophenol was then measured by continuous absorbance measurements at 400 nm. The initial linear slope of the generated curve was then used to calculate the activity with the use of a standard curve (absorbance versus *p*-nitrophenol concentration) and the dilution factors.

HPLC was used for analysis of the metabolites and substrates. Samples of the fermentation liquid were centrifuged (16,000 × *g*) in 2-mL Eppendorf tubes at 14,000 rpm for 5 min (Z 160 M; HERMLE Labortechnik, Wehingen, Germany). The supernatant was filtered using 0.2-μm filters, and the filtered samples were stored at -20°C. The sugar and glycerol concentrations were determined using a polymer column (Aminex HPX-87P; Bio-Rad Laboratories, Munich, Germany) at 85°C. MilliQ-water was used as an eluent, with a flow rate of 0.6 mL min^-1^. Ethanol, acetate, HMF (5-hydroxymethylfurfural) and furfural were analyzed using an Aminex HPX-87 H column (Bio-Rad Laboratories, Munich, Germany) at 60°C. The eluent was 5 mM H_2_SO_4 _with a flow rate of 0.6 mL min^-1^. The sugars, glycerol and ethanol were detected with a refractive index detector (Waters 2410; Waters, Milford, MA, USA) and acetate, HMF and furfural with a UV detector at a wavelength of 210 nm (Waters 2487; Waters, Milford, MA, USA).

### Calculations

The ethanol yield, *Y*_E/S_, was calculated on the basis of the total amount of fermentable sugars added to the SSCF, i.e., the sum of available glucose and xylose present in the pretreatment slurry, including monomers, oligomers and polymers (glucan and xylan fibers). The theoretical mass of glucose released during hydrolysis is 1.11 times the mass of glucan (due to the addition of water). The maximum theoretical ethanol yield is 0.51 g g^-1 ^on hexoses (as well as on xylose), and the fraction of the theoretical yield, Y*_E/S _obtained was calculated as Y*_E/S_= (*Y*_E/S_/0.51).

## Results

The aim of the current study was to investigate dual feeding, i.e., a combination of both substrate and enzyme feeding as a means to improve xylose utilization in SSCF of pretreated wheat straw. The basic approach was to accomplish a low but nonzero glucose concentration by controlled addition of substrate and enzymes throughout the course of the fermentation. The substrate addition enabled good mixing by preventing a too high viscosity, and thereby a larger amount of totally added WIS than possible in a batch process could be examined.

### Reference fed-batch SSCF

**Figure 1:**
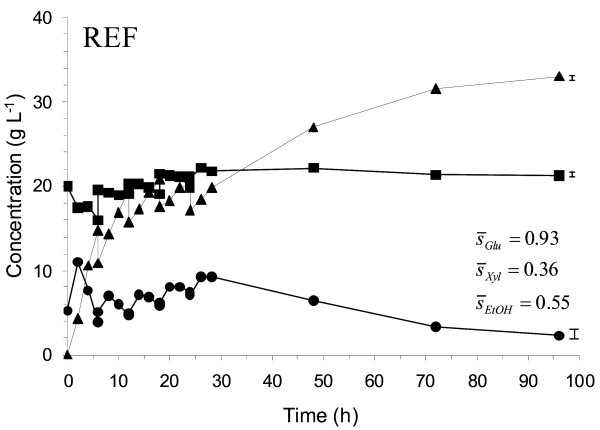
**Measured concentrations during duplicate reference fed-batch simultaneous saccharification and co-fermentation (SSCF) of wheat straw (8%-11% WIS) showing glucose (black circle), xylose (black square) and ethanol (black triangle)**. No enzyme feed was used; all enzymes were added initially. The mean standard deviation () is given for the concentration profile and illustrated graphically at the right end of each profile.

The substrate addition scheme used was the same in all the SSCF experiments. Substrate was added after 6, 12, 18 and 24 h. A standard fed-batch SSCF (starting at 8% and finishing at 11% WIS, with all enzymes initially added to the broth) was carried out as a reference (Figure 1). The reference experiments gave a xylose conversion of about 40% and an ethanol yield of 0.31 g g^-1^. Due to severe stirring problems caused by the high viscosity, it was not possible to perform a batch reference at a WIS loading of 11%. However, comparisons of batch SSCF and fed-batch SSCF of wheat straw using TMB3400 have previously been made [10] at both 7% and 9% WIS loading, and the fed-batch process gave significant improvements in both xylose conversion and overall ethanol yield (based on all available sugars).

### Fed-batch SSCF with enzyme feeding

**Figure 2:**
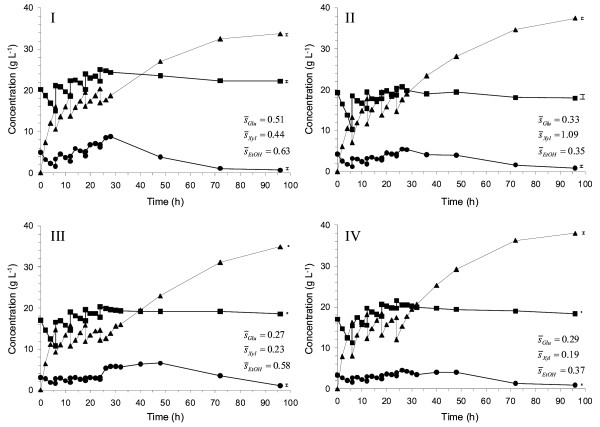
**Measured concentrations during duplicate fed-batch SSCF of wheat straw (8%-11% WIS) showing glucose (black circle), xylose (black square) and ethanol (black triangle) when using different enzyme feed strategies: (I) Feed profile A, (II) Feed profile B, (III) Feed profile C, (IV) Feed profile D**. The mean standard deviation () is given for the concentration profile and illustrated graphically at the right end of each profile.

Four different enzyme feed profiles (Table 2) were investigated in SSCF of wheat straw. All experiments were carried out in duplicate and showed excellent reproducibility as indicated by the standard deviations in Figures 1 and 2. A small initial enzyme addition was used in all enzyme feed strategies to decrease the initial viscosity and eliminate major stirring and mass transfer problems. The experimental results clearly showed the significant potential to increase the xylose consumption by combining substrate and enzyme feeding. In the best case tested (feed profile B, SSCF II), the xylose conversion increased from 40% to 50%. At the same time, the ethanol yield increased from 0.31 to 0.35 g g^-1^, and the final ethanol concentration increased from 33 to 38 g L^-1 ^(Table 3). However, not all profiles gave improvements.

**Table 2:** Enzyme feed strategies for fed-batch simultaneous saccharification and co-fermentation of wheat straw (8%-11% WIS)^a^

Time of addition (h)	Feed profile A (amount enzyme)	Feed profile B (amount enzyme)	Feed profile C (amount enzyme)	Feed profile D (amount enzyme)
Initial addition	2/6	1/5	1/5	1/5
6	1/6	1/10	-	-
12	1/6	1/10	-	-
18	1/6	1/10	-	-
24	1/6	1/10	1/5	1/5 (+ 2 g/L yeast)
32	-	-	1/5	1/5
36	-	1/5	-	-
40	-	-	1/5	1/5
48	-	1/5	1/5	1/5

^a^In all feed profiles, the same total enzyme load and the same substrate feed were used. Substrate was added after 6, 12, 18 and 24 h.

**Table 3:** Summary of duplicate fed-batch simultaneous saccharification and co-fermentation of wheat straw (8%-11% WIS)

Fed-batch SSCF	Enzyme feed profile	Xylose consumption^a ^(%)	Xylitol formation^b ^(%)	Final ethanol concentration (g L^-1^)	Ethanol yield (g/g)	Ethanol yield^c ^(%)
I	A	38	12	33.7	0.31	61
II	B	50	8	37.4	0.35	68
III	C	46	6	34.9	0.33	65
IV	D^d^	49	9	38.0	0.35	69
REF	No feed	40	7	33.0	0.31	61

In all experiments, a total enzyme load of 36 FPU (g glucan)^-1 ^and a yeast load of 4 g L^-1 ^were used.

^a ^Related to total amount of available xylose.

^b ^Related to consumed xylose.

^c ^Corresponding to the maximum theoretical yield on total available sugars.

^d ^An extra yeast addition (2 g L^-1^) was made at t = 24 h (see last paragraph in the results section).

In the initial profile tested, the enzymes were fed along with the substrate (Case A, Table 2). This feed profile did not give any significant increase in ethanol yield or final ethanol concentration compared with the reference fed-batch case (Table 3). The glucose concentration profile was also very similar to the reference SSCF (Figure 1 and 2), which means that a slower glucose release rate is needed to obtain lower glucose concentrations during the fermentation.

Two other enzyme feed profiles were therefore assessed with this objective. In Case B, the enzyme additions were made smaller but continued during a longer time compared with Case A, whereas in Case C, the enzyme additions were not started until the last substrate addition was made. Case B resulted in an increase of both the ethanol yield and the final ethanol concentration (Figure 2, II). The glucose concentration was in this case lower throughout the SSCF in comparison to Case A. In Case C, a slight increase in ethanol yield and final ethanol concentration resulted compared with the reference SSCF (Figure 2, III), but not as much as in Case B. This was likely due to the high glucose levels after 24 h of SSCF. Nevertheless, looking at the glucose concentration between 0 and 24 h of SSCF, feed profile C seemed promising. However, when the enzyme additions are initiated at 24 h, the glucose release rate is apparently too high after the enzyme addition.

Another way of decreasing the glucose concentration is to increase the consumption of glucose by adding more yeast. To investigate whether it was possible to increase the xylose utilization by lowering the glucose concentration in this manner, experiments were conducted in which additional yeast was added after 24 h (Case D). As shown in Figure 2 (IV), the glucose concentration could now be maintained at a low level also after 24 h, and, as expected, this resulted in a higher ethanol yield and a higher final ethanol concentration compared with Case C. However, neither the ethanol yield nor the final ethanol concentration was significantly higher than in Case B (Figure 2, II).

## Discussion

SSCF is a feasible process option for co-fermentation of glucose and xylose, because it allows a slow, constant release of glucose throughout the process that is beneficial for xylose uptake by xylose-fermenting strains of *S. cerevisiae *[10,19,22]. However, there is an upper level of WIS content due to the increasing viscosity with an increasing WIS content. This may result in severe mixing problems. By making a fed-batch SSCF, i.e., by adding the WIS gradually, a higher total WIS loading in the reactor is possible [10].

In the current study, a combination of fed-batch SSCF and enzyme feeding was examined for the first time to improve xylose utilization at a high WIS loading level. The experimental results showed a significant increase in xylose consumption as well as increased ethanol yield and final ethanol concentration when comparing the process with enzyme and substrate feeding to the process with only substrate feeding. The current study builds on previous work to lower and/or control the glucose concentration by designing the SSCF in different ways. Three principal designs have previously proven to facilitate xylose utilization in SSCF of lignocellulosic materials: fed-batch (substrate feed), prefermentation and controlled enzyme feeding [10,21,22]. The prefermentation concept was not included in the current study because of stirring issues caused by the high WIS loading. Furthermore, prefermentation has its major advantage in a batch process SSCF in which the pretreated material is rich in free hexoses, such as spruce.

The ethanol yield in SSF typically decreases rather rapidly with increasing WIS content as discussed by Olofsson et al. [5]. Obviously, the optimum WIS content in the process is determined by the balance between distillation cost reductions at higher final ethanol titers and a decreased ethanol yield (i.e., a higher cost of the raw material) at higher WIS loading levels. It is not possible to make a strict comparison of yields to previous studies, owing to differences in raw materials and WIS loading levels. However, an approximate comparison between the present results and the results on fed-batch SSCF using wheat straw (6%-9% WIS) by Olofsson et al. [10] shows that a clear improvement of the xylose consumption, from 37% to 50%, despite a much higher WIS content, was obtained in the present study (8%-11% WIS). Moreover, the ethanol yield could be maintained at the same level, around 70% of theoretical, even at this higher WIS content. The combination of enzyme and substrate feed therefore shifts the process optimum to higher WIS loadings in SSCF of pentose-rich materials, because it allows increasing the WIS content with only a low decrease in ethanol yield.

The results in Table 3 and a comparison of SSCF II, III and IV with the reference SSCF make it evident that the increase in ethanol yield is not only explained by the increased xylose uptake. One can estimate, using the theoretical yield 0.51 g g^-1^, that 35% to 55% of the increase in ethanol concentration comes from the additional xylose taken up. Because the by-product formation did not decrease, an improvement in glucan conversion also resulted from the enzyme feeding.

The positive effect on the xylose conversion by a low glucose concentration can be understood from the very different affinities for glucose and xylose in the sugar transport system of *S. cerevisiae*. Because the affinity for glucose is around 200 times higher than for xylose [14], it is evident that high glucose concentration will affect the xylose utilization negatively. However, as shown also in this study, keeping a low glucose concentration is apparently not enough, because the xylose uptake is reduced over time in all the SSCF experiments. In the present SSCF study, there was as much as 15-20 g L^-1 ^xylose left in the reactor at the end, and there was in fact very little xylose uptake after about 30 hours of fermentation. In a previous work with spruce [22], it was shown possible to consume xylose down to a concentration below 1 g L^-1^, indicating that the problem is not a too low xylose concentration *per se*. Glucose is known to enhance xylose uptake at low but nonzero concentrations, and a certain intracellular glycolytic flux thus has to be maintained for efficient xylose utilization [15,16]. Possibly this could explain a decreasing xylose uptake rate in the SSCF. However, the complete stop of the xylose uptake cannot be explained in this manner, because glucose is continuously released throughout the SSCF. An explanation for this could instead be a general downregulation of sugar uptake and metabolic activity of the yeast caused by a gradually increasing inhibition from the hydrolyzate medium. This hypothesis was tested by addition of fresh yeast after 24 hours fermentation. The resulting decrease in glucose concentration and the concomitant increase in ethanol concentration (compare SSCF III and SSCF IV in Figure 2) clearly showed that the yeast was active. However, the xylose uptake did not increase even after addition of fresh yeast, which suggests a more specific inhibition on the xylose metabolism or the xylose uptake. Given that the xylose uptake and metabolism is functioning for the first 30 hours, it is reasonable to believe that the tentative inhibitor(s) is one (or several) compound(s) formed during the SSCF. Possibilities include xylitol, glycerol, ethanol and/or other alcohols formed by reduction of other aldehydes, e.g., furans. Although several studies have been carried out concerning kinetics of the xylose reduction in yeast [31-33], comprehensive reports on the inhibition of the pathway are missing. One tentative target of inhibition is the XR (xylose reductase). In the current study, the xylitol concentration was found to stay below 2 g L^-1 ^throughout the SSCF, which could indicate a threshold level. However, the strain TMB3400 has in other media been able to produce much higher xylitol concentrations (up to 10 g L^-1^), which strongly suggests that xylitol is not the inhibitor. XR is known to catalyze the reduction of several compounds apart from xylose in lignocellulosic hydrolysates, e.g., furfural and HMF to furan-2,5-dimethanol (FDM) and 2-furanmethanol (FM) [34], and these compounds may affect the activity of XR. Evidently, more studies are needed to find the reason why xylose uptake stops in the later part of the SSCF. A specific inhibition of the heterologous xylose pathway introduced may be a point of concern even for a yeast strain which *per se *is tolerant.

One option to increase xylose utilization is to introduce other xylose transporters into the yeast. Recent results have shown that by introducing the xylose facilitator *GXF1 *from *Candida intermedia *in *S. cerevisiae *[35], the xylose uptake could be improved significantly in SSCF (Fonzeca and Olofsson, unpublished data). However, the ethanol yield was not increased. Instead, the additional xylose taken up resulted in a higher yield of xylitol and glycerol, showing that an improved uptake system is not by itself sufficient. The XR from *Pichia stipitis*, which is expressed in TMB3400, can use either NADPH or NADH as a cofactor. The dual cofactor dependence of XR may prevent complete regeneration of NAD^+^, which is needed for the XDH (xylitol dehydrogenase) reaction. This is a well-known problem and may explain the excretion of xylitol, although other reductases, notably *GRE3 *[36], also plays a role in this. Important progress has recently been made to obtain a XR-XDH pathway which is more cofactor balanced in itself [37].

In conclusion, our study has shown that by using both enzyme and substrate feeding, the xylose conversion in SSCF could be improved from 40% to 50% in comparison to regular fed-batch, which should be noted is as such already superior to the batch SSCF. Furthermore, by careful combination of different feeding strategies, we have shown that it is actually possible to increase the final WIS content to 11% in the SSCF and still obtain an ethanol yield of 0.35 g g^-1 ^of fermentable sugars.

## Competing interests

The authors declare that they have no competing interests.

## Authors' contributions

KO participated in the design of the study, performed the experimental work and wrote the manuscript. BP performed the experimental work and commented on the manuscript. GL participated in the design of the study and commented on the manuscript. All authors contributed to the scientific discussion throughout the work and have read and approved the final manuscript.
